# The 3-Steps Approach for Lumbar Stenosis with Anatomical Insights, Tailored for Young Spine Surgeons

**DOI:** 10.3390/jpm14090985

**Published:** 2024-09-16

**Authors:** Giuseppe La Rocca, Gianluca Galieri, Edoardo Mazzucchi, Fabrizio Pignotti, Vittorio Orlando, Simona Pappalardo, Alessandro Olivi, Giovanni Sabatino

**Affiliations:** 1 Institute of Neurosurgery, Fondazione Policlinico Universitario A. Gemelli IRCCS, Catholic University, 00168 Rome, Italy; giuseppe.larocca@policlinicogemelli.it (G.L.R.); alessandro.olivi@policlinicogemelli.it (A.O.); giovanni.sabatino@policlinicogemelli.it (G.S.); 2 Neurosurgical Training Center and Brain Research, Mater Olbia Hospital, 07026 Olbia, Italyfabrizio.pignotti@materolbia.com (F.P.); 3 Department of Neurosurgery, IRCCS Regina Elena National Cancer Institute, 00144 Rome, Italy; 4 Department of Neurosurgery, Mater Olbia Hospital, 07026 Olbia, Italy; 5 Department of Anatomical Pathology, Giovanni Paolo II Hospital, 97100 Olbia, Italy

**Keywords:** lumbar stenosis, 3-step, trapezoid, decompression, young surgeon

## Abstract

**Background/Objectives**: Lumbar decompression surgery for degenerative lumbar stenosis is an intervention which addresses a degenerative condition affecting many patients. This article presents a meticulous three-phase surgical approach, derived from our clinical experiences and intertwining anatomical insights, offering a nuanced perspective tailored for the educational needs of young spinal surgeons. **Methods**: Six hundred and eighty-seven patients who underwent lumbar decompression surgery at a single institution were included in the present study. A retrospective analysis of patient demographics and surgical techniques was performed. All surgeries were performed by a consistent surgical team, emphasizing uniformity in approach. The surgical technique involves a meticulous three-phase process comprising exposure and skeletal visualization; microscopic identification and decompression; and undermining of the spinous process base and contralateral decompression. **Results:** Presenting results from 530 patients, the study examines demographic characteristics, health profiles, operative details, complications, and clinical assessments. The three-phase approach demonstrates low complication rates, absence of recurrences, and improved clinical outcomes, emphasizing its efficacy. **Conclusions**: The three-phase surgical approach emerges as a valuable educational tool for both novice and seasoned spinal surgeons. Rooted in anatomical insights, the structured methodology not only caters to the educational needs of young surgeons, but also ensures a standardized and safe procedure. The emphasis on tissue preservation and anatomical points aligns with current trends toward minimally invasive techniques, promising enhanced patient outcomes and satisfaction.

## 1. Introduction

Lumbar spinal stenosis (LSS) is characterized by the narrowing of the spinal canal. Epidemiological studies underscore the prevalence of LSS, considering shifts in demographics and the increased occurrence of age-related musculoskeletal disorders [1]. Over time, this condition has incurred substantial healthcare costs, especially with the rise in life expectancy [2]. This article aims to incorporate historical perspectives into our experience-based surgical three-step methodology for the surgical treatment of lumbar stenosis, offering insights into its characteristics and advantages over traditional surgical approaches in an organized and schematic manner.

### Epidemiology and Pathophysiology

The etiology of lumbar spinal stenosis (LSS) is commonly classified as either acquired (degenerative) or congenital, and affects around 103 million individuals worldwide [3].

The degenerative form becomes more prevalent with age [4]. Anatomically, degenerative LSS is categorized into central, lateral, and foraminal stenosis, with a higher occurrence observed at the L4–5 level [4]. The main causes include thickening of the ligamentum flavum (associated with ligament buckling due to disc height reduction) and facet joint hypertrophy (osteoarthritis), or a combination of both [4,5].

Congenital lumbar spinal stenosis (LSS) arises from disruptions in the growth of the dorsal parts of vertebrae during prenatal or early infancy or premature fusion of the posterior elements [6,7]. Distinctive features include short pedicles and metabolic syndromes like Paget disease and epidural lipomatosis, typically associated with corticosteroid excess [4,8].

The pathophysiology of symptomatic LSS is not fully understood. In the early 1990s, Porter and Ward proposed the “double-crush” theory, suggesting that neural structures in symptomatic patients are often compressed by at least two anatomical areas, occurring at multiple levels or in both central and foraminal locations. Recent investigations have further linked the specific clinical symptoms of LSS to a combination of mechanical compression and disruption of blood flow, involving either arterial ischemia or venous congestion within the cauda equina or individual nerve root [9,10].

## 2. Materials and Methods

### 2.1. Study Design

A retrospective study was conducted, including 687 patients who underwent surgery for lumbar spinal stenosis at Mater Olbia Hospital between July 2019 and October 2022. Patients eligible for inclusion were 18 years of age or older, had a confirmed diagnosis of lumbar spinal stenosis, and had not responded to at least 6 months of conservative treatments. These treatments included activity modification, medication, epidural steroid injections, as well as a structured physical therapy program focused on diet, exercise, and weight loss. All patients required a follow-up period of at least 12 months.

### 2.2. Data Collection

Patient data were gathered both pre- and post-surgery to evaluate factors such as lower back pain, leg pain, quality of life, and psychosomatic aspects. Additional information, including patient demographics like age, weight, body mass index (BMI), smoking habits, and other risk factors, was also recorded. All procedures were carried out by the same two surgeons to maintain consistency in the surgical approach, and a standardized technique was applied across all patients.

### 2.3. Follow-Up

Patients received a follow-up evaluation one month after surgery, which included lumbosacral X-rays. Subsequent assessments were carried out annually, leading to a total follow-up duration ranging from 1 to 4 years for each patient.

### 2.4. Additional Data Collection

The length of surgery and hospital stay was recorded for each patient. Intraoperative and postoperative complications were tracked and analyzed. Recurrence rates of LSS and the potential for subsequent stenosis-related instability were assessed throughout the follow-up period. All participants provided written informed consent, and the study received prior approval from the local Ethics Committee under protocol number 276/2020/CE.

## 3. Surgical Technique

### 3.1. Anatomical Consideration

Each lumbar vertebra boasts distinctive features: (1) the spinous process extends backward, offering anchorage for muscles and ligaments; (2) the transverse processes provide attachment points for muscles and contribute to stability; (3) the articular processes, both superior and inferior and joined through the pars interarticularis, form joints with neighbouring vertebrae, influencing the range of spinal movement; (4) the pedicles connect the vertebral body to the lamina, essentially acting as a bridge; and (5) the vertebral body bears the weight, serving as the structural backbone [11,12,13].

Nestled between the vertebral bodies lies the intervertebral disc, which comprises the outer annulus fibrosus and the inner nucleus pulposus, collectively acting as a shock absorber and facilitator of movement [11,14].

Within the vertebral canal resides the yellow ligament, providing both support and flexibility. Its elastic nature helps limit excessive flexion, contributing to the spine’s stability [11,12].

### 3.2. Posterior Surgical Lumbar Trapezoid: Anatomical Landmarks

**Figure 1:**
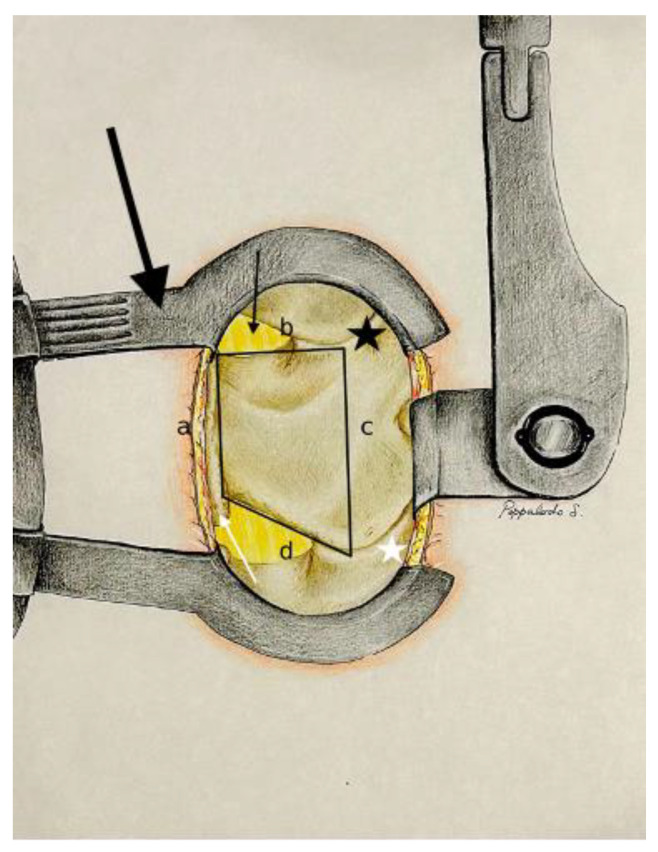
Exposition after Caspar distractor position and skeletalization of homolateral spinous process and homolateral lamina (Step 1). Dentification of the posterior surgical trapezoid: (**a**) from caudal to cranial point of the base of the spinous process; (**b**) from the cranial point of the base of the spinous process to the medial third of the superior articular process (black star); (**c**) from the medial third of the superior articular process to the medial third of the inferior articular process (white star); (**d**) from the caudal point of the base of the spinous process to the medial third of the inferior articular process. Base of the spinous process (white arrow); yellow ligament (black arrow); Caspar distractor (big black arrow).

The posterior surgical lumbar trapezoid (Figure 1) is precisely demarcated by four key anatomical landmarks, creating a defined space for targeted surgical intervention.

- The caudal margin of the spinous process base is situated at the lower base of the spinous process, and marks the inferior limit of the trapezoidal space.
- The cranial margin of the spinous process base is located at the upper base of the spinous process, and establishes the superior boundary of the trapezoid.
- The medial margin of the superior articular process is defined by the inner edge of the superior articular process, and marks the medial limit on the superior aspect of the trapezoid.
- The medial margin of the inferior articular process is found along the inner edge of the inferior articular process, and represents the medial boundary on the inferior aspect of the trapezoid.

In our opinion, understanding the significance of each landmark equips aspiring spine surgeons with a navigational tool, providing a clear and precise surgical field and minimizing the risk of unintentional damage to neighboring structures, as referred to in the following results.

### 3.3. Pre-Operative Evaluation, Positioning, and Surgical Level Localization

**Figure 2:**
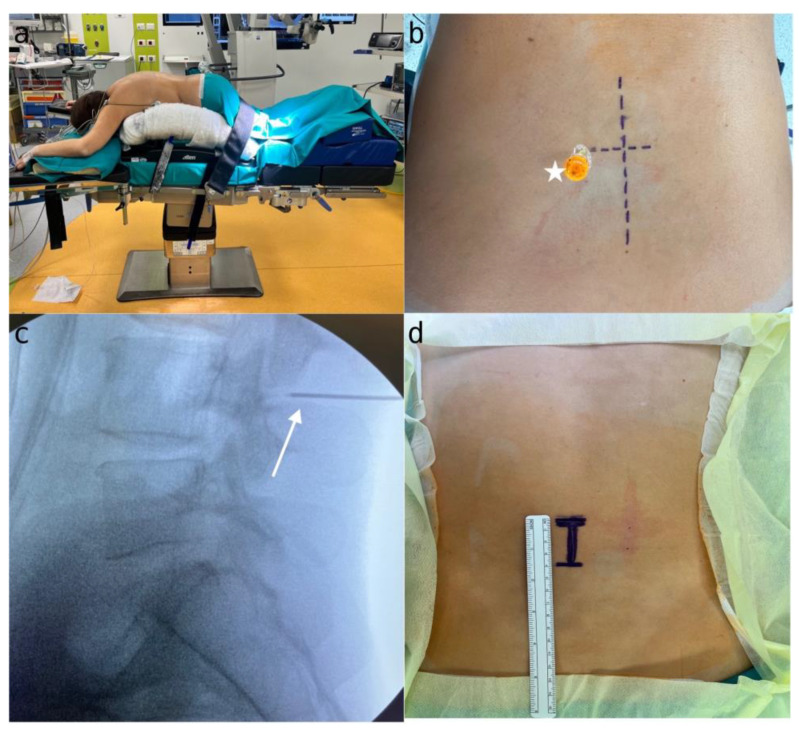
(**a**) Patient positioning on the Wilson frame and setup of the operating room; (**b**) spinal needle (white star) placed laterally on the opposite articular process of the surgical site, with the long line indicating the midline and the short line marking the L4L5 level; (**c**) X-ray confirmation of spinal needle placement (white arrow) to ensure correct targeting of the L4 level; (**d**) skin incision marked, with one-third extending superiorly and two-thirds inferiorly from the point of interest, measuring approximately 4 cm in length.

The pre-operative evaluation process (performed in order to avoid error in patient selection, to confirm the correct level and side, the use of preoperative antibiotics, the type of anesthesia, and to avoid the risk of thromboembolism), the correct position on the operative field (Figure 2a), and our X-ray method to identify the involved level (Figure 2b–d) can be referred to in the article cited later [11].

## 4. Three Step Approach for Lumbar Spinal Stenosis

### 4.1. Step 1: Exposure and Skeletal Visualization

**Figure 3:**
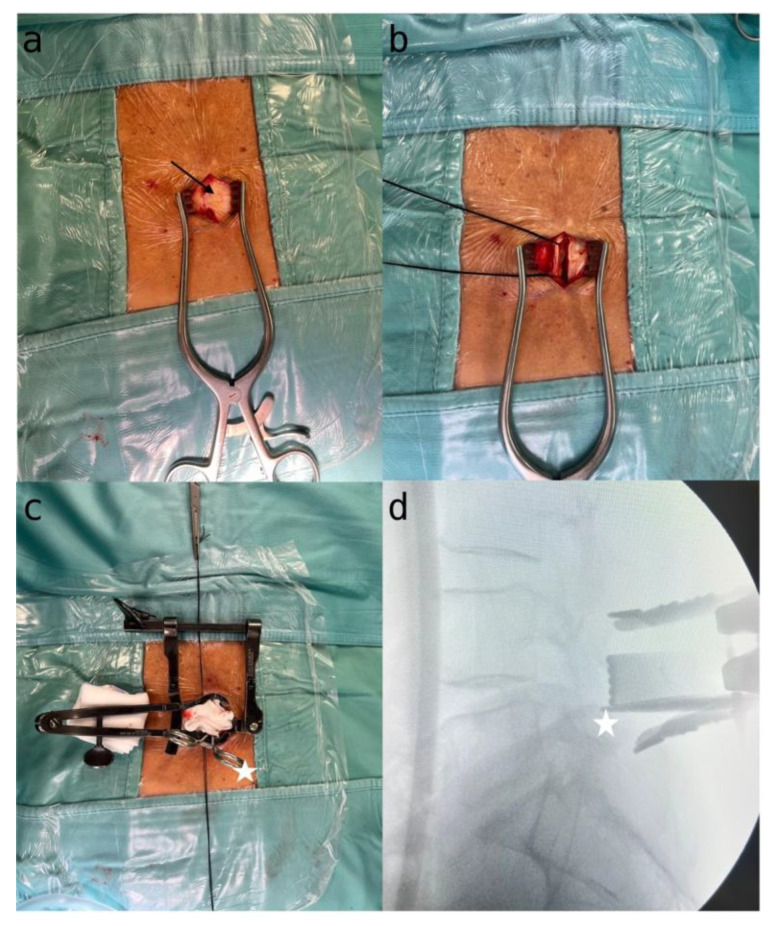
(**a**) Exposure of the skin incision and the muscular fascia (black arrow); (**b**) handling of the muscle fascia; (**c**) skeletonization of the homolateral lamina and positioning of a Caspar distractor. Klemmer forceps (white star) placed beneath the lamina for X-ray verification; (**d**) X-ray confirmation of the Caspar distractor’s accurate placement at the correct level.

First, a midline skin incision of around 3–3.5 cm in length is made, to allow for a focused, minimalistic surgical approach. A paramedian linear incision is then made in the muscle fascia on the most symptomatic side, providing targeted access to the affected area (Figure 3a).

The muscle fascia on the symptomatic side is carefully dissected while preserving muscle integrity to ensure optimal exposure. A silk thread is used to suspend the dissected fascia, allowing for clear and unobstructed visualization of the surgical field (Figure 3b).

Precise skeletonization of the lamina on the affected side is performed, ensuring clear identification and access to the target vertebral structures. A Caspar or Scoville distractor is used to enable controlled separation and better visualization (Figure 3c).

To confirm the correct vertebral level, a Penfield dissector or Klemmer forceps are used, placed at the lower edge of the lamina, with radioscopic imaging to ensure precision and alignment during the procedure (Figure 3d). This step is crucial for preserving the integrity of surrounding structures and guiding subsequent surgical actions.

### 4.2. Step 2: Microscopic Identification and Decompression

Using an intraoperative microscope, the surgical field is magnified to enhance precision during detailed procedures. The anatomical lumbar trapezoid is utilized to locate key landmarks, including the midline between the upper and lower points of the spinous process base, as well as the medial aspects of both the superior and inferior articular processes (Figure 1).

**Figure 4:**
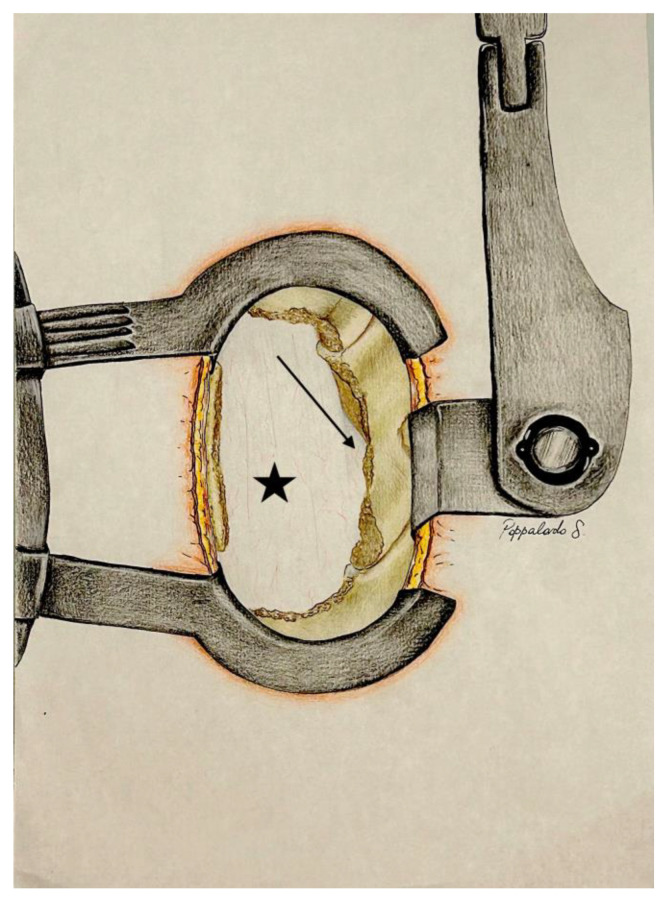
Exposure following the removal of the homolateral lamina, one third of the superior and inferior articular process, and the yellow ligament (Step 2). Dural sac (black star); nerve root (black arrow).

A Kerrison rongeur or a high-speed drill with a diamond-tipped burr is used to carefully remove a portion of the lamina within the trapezoid, stopping at the medial borders of the superior and inferior articular processes, which remain intact. Full visualization of the yellow ligament, now separated from the lamina, is achieved. The ligament is then gently lifted and detached by positioning the Kerrison forceps under the lower portion of the lamina. The forceps are moved in a supero-caudal direction, then rotated infero-caudally to carefully release the ligament from the base of the spinous process. Finally, using Weil forceps, the ligament is carefully removed laterally towards the articular process, allowing direct visualization of the epidural fat and enabling homolateral decompression of the spinal cord (Figure 4).

### 4.3. Step 3: Undermining of the Spinous Process Base and Contralateral Decompression

With the aim of a spatula or Penfield dissector, a high-speed drill or Kerrison forceps are used to meticulously remove a small section of the spinous process base. Careful adjustments are made to the operating table, tilting it approximately 15–30 degrees towards the side that requires decompression. Simultaneously, the microscope is fine-tuned by lowering it around 5 cm and tilting it 15–20 degrees. These precise adjustments are essential for achieving an enhanced view of the lower part of the spinous process and the contralateral area.

**Figure 5:**
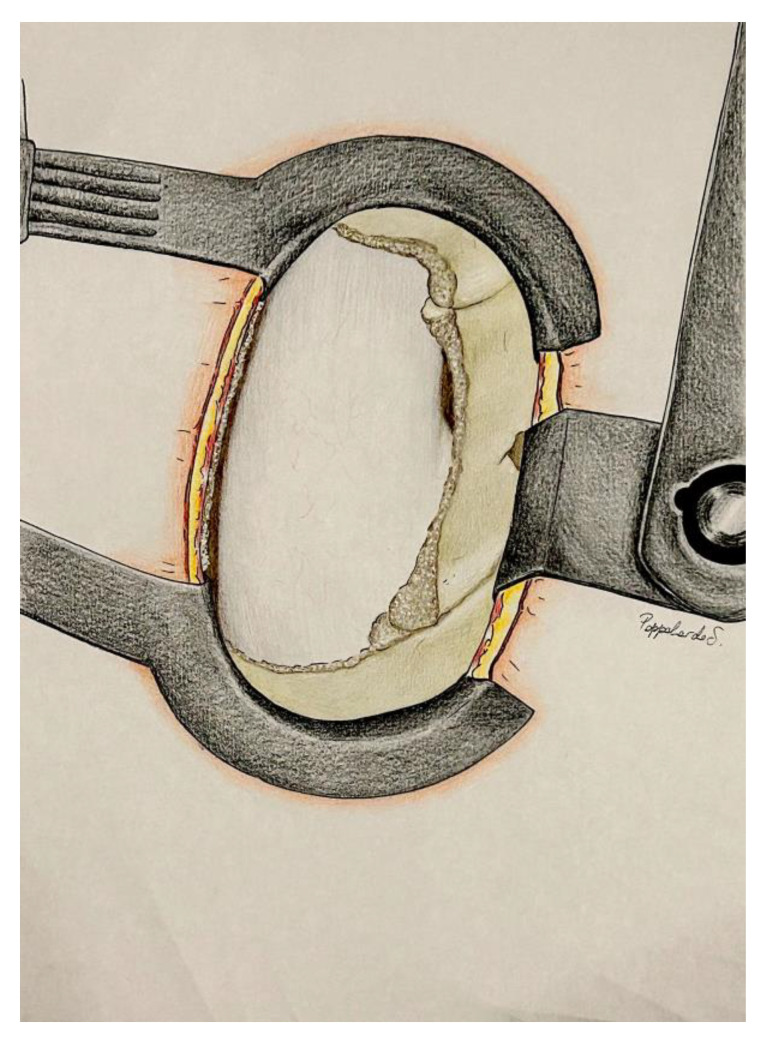
After table tilt and microscope adjustment, undermining of the spinous process base and contralateral decompression (STEP 3) removing contralateral yellow ligament.

Under direct visualization, the removal of the entire yellow ligament from the contralateral side is carried out, ensuring thorough and precise decompression of the contralateral side (Figure 5).

This strategic unilateral approach, based on anatomical insight, ensures an optimal bilateral decompression of the dural sac, under direct visualization, marking a significant achievement within our tailored surgical approach.

## 5. Results

### 5.1. Study Population 

Between July 2019 and October 2022, a total of 687 patients underwent surgical treatment for lumbar spinal stenosis at Mater Olbia Hospital. Of these, 157 patients were excluded from the study due to having a follow-up period of less than one year. Ultimately, 530 patients met the inclusion criteria and were analyzed. Clinical and demographic data for these patients can be found in Table 1.

### 5.2. Operative Details

A total of 789 lumbar levels were operated upon, distributed across specific vertebrae: L5S1 (87), L4L5 (360), L3L4 (234), L2L3 (92), L1L2 (13), D11D12 (1), D10D11(1), and D9D10 (1) (Table 2). Symptomatic sides were prevalent on the right in 294 cases and on the left in 236 cases. Surgical metrics included an average hospital stay of 2.64 days (±0.78), with a mean surgical duration of 47 min (±19.4). The mean length of the surgical incision was 4.61 cm (±1.68), and subfascial surgical drainage was utilized in 46 cases; this was removed during the first post-operative day without complications. 

### 5.3. Complications

Intraoperatively, complications included thirty-one cases (5.85%) of cerebrospinal fluid (CSF) leaks and one instance (0.19%) of atrial fibrillation. Postoperative complications comprised three subfascial hematomas (0.57%), one occurrence of amaurosis in the left eye (0.19%), one case of postoperative anemia (0.19%), one thromboembolism (0.19%), eight surgical site infections (1.52%), and twenty-seven cases (5.1%) with persistent or exacerbated symptoms despite the absence of radiological stenosis recurrence (Table 3).

### 5.4. Pre and Postoperative Clinical Assessment

Clinical evaluations, pre and postoperatively, utilized the Oswestry Disability Index (ODI), Visual Analogue Scale (VAS) for back and leg pain, and the Euro Quality 5 Dimensions (EQ-5D). The mean preoperative ODI was 52.2 (SD 18.6), decreasing to 24.7 (SD 18.7) postoperatively. VAS scores showed a reduction in back pain from a preoperative mean of 8.1 (SD 1.37) to a postoperative mean of 2.2 (SD 2.3), and in leg pain from 8.1 (SD 1.4) to 3.1 (SD 2.5). The EQ-5D demonstrated a preoperative mean of 0.377 (SD 0.211), improving to a postoperative mean of 0.684 (SD 0.233) (Table 4).

### 5.5. Measurement

During our surgical observations, which included both physical measurements and images captured with the microscope (Microscope Leica M530 OHX, Leica Microsystems, Wetzlar, Germany), we examined the dimensions of the posterior surgical lumbar trapezoid (Table 5).

We found that the length of the first side, extending from the cranial to the caudal part of the base of the spinous process, averaged about 16.6 mm with a variation of ±2.02 mm. The second side, which stretches from this cranial base to the superior articular process, measured approximately 20.3 mm, with a standard deviation of ±1.28 mm. The third side, running from the superior articular process to the inferior articular process, averaged 26.5 mm, showing a range of ±2.06 mm. Lastly, the fourth side, from the inferior articular process back to the caudal part of the base of the spinous process, had an average length of 21.5 mm, with a deviation of ±1.86 mm.

In addition to these measurements, we also assessed the area of bone removed from the lamina, which roughly corresponded to the trapezoidal shape we studied. The average area of this excised bone was found to be 437.4 mm^2^, with a variability of ±40.4 mm^2^.

## 6. Discussion

### 6.1. Impact of Surgical Approach 

In a randomized clinical trial by Hermansen et al., the researchers investigated three minimally invasive techniques (unilateral laminotomy with crossover, bilateral laminotomy, and spinous process osteotomy) for posterior lumbar decompression in 437 patients affected with LSS. There were no differences in clinical outcomes or complication rates found among the three minimally invasive posterior decompression techniques, except a longer duration of the surgical procedure in the bilateral laminotomy group [15].

In a review by Overdevest et al., a total of 733 patients were included. Three studies (173 participants) compared unilateral laminotomy for bilateral decompression with conventional laminectomy; four studies (382 participants) compared bilateral laminotomy with conventional laminectomy; and four studies (218 participants) compared split-spinous process laminotomy with conventional laminectomy. The review found that different posterior decompression techniques and the conventional laminectomy had similar effects on functional disability and leg pain. However, perceived recovery at final follow-up was better in patients who underwent bilateral laminotomy. Unilateral laminotomy for bilateral decompression and bilateral laminotomy showed fewer cases of iatrogenic instability, though the incidence was low in both cases. Postoperative low back pain severity was less following bilateral laminotomy and split-spinous process laminotomy compared to conventional laminectomy, but the difference was not clinically significant. There was no evidence of differences in complication rates, procedure length, hospital stay duration, or postoperative walking distance between the different posterior decompression techniques [16].

Another trial by Rajasekaran et al. compared lumbar decompression techniques in 51 patients, finding no significant differences in outcomes at a 14.2-month follow-up [17]. Similarly, a separate study comparing conventional microsurgical decompression (100 patients) with full-endoscopic interlaminar decompression (92 patients) showed no notable differences at the 2-year mark [18].

While prospective observational studies highlight symptom improvement with minimally invasive decompression, comparative studies suggest no clear superiority over traditional methods concerning pain, disability, quality of life, or walking ability. However, minimally invasive techniques are associated with significantly shorter postoperative hospital stays [16,19].

### 6.2. Comparation with Others Approachs

#### 6.2.1. Comparison with Open and Traditional Approaches

Traditional open decompressive laminectomy has long been the gold standard for LSS treatment. While effective in relieving neural compression, it is often associated with significant muscle dissection, increased blood loss, longer operative times, and extended hospital stays [15]. In contrast, our three-step approach minimizes muscle trauma through a focused midline incision and skeletal visualization, resulting in a shorter average surgical duration of 47 min and an average hospital stay of approximately 2.64 days. These metrics are comparable to or better than those reported in conventional open surgeries, which typically exhibit longer operative times and hospitalization periods [16,17].

#### 6.2.2. Microscopic Unilateral vs. Bilateral Decompression

Microscopic approaches, including unilateral hemilaminectomy and bilateral laminotomy, offer enhanced visualization and reduced soft tissue disruption. Unilateral hemilaminectomy, similar to our approach, allows for targeted decompression with potentially fewer complications related to spinal instability [18]. Bilateral decompression techniques, while providing comprehensive neural decompression, often entail longer operative times and a higher risk of iatrogenic instability [16,19]. Our approach, which incorporates elements of unilateral decompression with strategic anatomical landmark identification, achieves effective bilateral decompression without the extended operative times or increased instability risks associated with bilateral laminotomy [20].

#### 6.2.3. Endoscopic and Microendoscopic Techniques

Endoscopic and microendoscopic approaches represent the frontier of minimally invasive spine surgery, offering benefits such as reduced postoperative pain, shorter recovery times, and minimal scarring [21]. However, these techniques require specialized equipment and extensive training, potentially limiting their widespread adoption. Additionally, the learning curve associated with endoscopic methods can impact surgical efficiency and outcomes in the initial phases of implementation [22]. Our three-step approach, while not as minimally invasive as endoscopic techniques, provides a balance between surgical precision and practicality, making it more accessible for surgeons without specialized endoscopic training.

### 6.3. Predictor of Outcome

The influence of age on postoperative outcomes is still a matter of debate. While some studies argue that older age, even with common health issues, does not reliably predict surgical success, others suggest a connection between advanced age and more severe postoperative symptoms [20,21]. Gender does not seem to play a significant role in surgical outcomes, although occasional reports hint at better long-term results in males. Obese individuals benefit from decompressive surgery, but not to the same extent as those with normal weight [22,23]. Preoperative depression has emerged as a significant predictor of more severe postoperative pain, disability, and reduced walking ability [24,25].

Smoking patients benefit from surgery, albeit not as much as nonsmokers [26]. Shorter symptom duration is linked to a higher likelihood of successful outcomes [27]. Patients with preoperative numbness often experience postoperative residual leg pain and numbness. Severe preoperative back pain, especially if worse than leg pain, strongly predicts a worse outcome. While patients with more severe preoperative disability may show greater postoperative improvement, they express less satisfaction with pain, function, and quality of life.

### 6.4. Surgical Complications

Perioperative complications (5.4% to 14%) and postoperative complications (8.2% to 18%) contribute to an overall mortality rate of 0.3% to 0.5% [28]. Major medical complications (3.1%) are more commonly associated with increased comorbidity [29]. The rate of general complications becomes higher with age, poorer ASA risk status, and increased intraoperative blood loss [30,31]. Notably, the rate of perioperative surgical complications does not linearly increase with age [31]. Common comorbidities often mentioned in the literature include osteoarthritis, cardiac disease, rheumatoid arthritis, and chronic pulmonary disease. The risk factors most strongly associated with an unfavorable outcome are preoperative complaints primarily related to low back pain, followed by preoperative comorbidities. Our findings reinforce the notion that while surgical technique is crucial, patient-specific factors also play a significant role in determining the success of decompressive surgery for LSS. 

Decompression procedures can lead to complications such as postoperative neurologic deficits, dural tears, cerebrospinal fluid fistulas, pseudomeningoceles, facet fractures, infections, and vascular injuries.

Accidental dural tears have been observed in 0% to 20.6% of patients undergoing traditional open decompression and in 0% to 12% of those opting for various minimally invasive techniques [32,33,34]. A meta-analysis indicates that the incidence rates for dural tears are 7.7% in traditional open procedures and 9.2% in minimally invasive approaches [35]. Factors such as older age, female gender, smoking, hypertension, and diabetes contribute to an elevated risk of experiencing a dural tear [36,37].

Spinal epidural hematoma is a rare complication of lumbar decompressive surgery, occurring in 0% to 3.3% of cases. Asymptomatic hematomas are found in 15% to 42.5% of postoperative MRIs [38,39,40]. When symptoms occur, the hematoma size is approximately twice as large as in asymptomatic cases [41]. These hematomas can extend beyond the decompressed area, both upward and downward, as well as to the opposite side. Importantly, the risk of symptomatic postoperative epidural hematoma is not increased with multilevel surgery [42].

Reoperation rates for recurrent stenosis or degenerative instability increase over time: 3–10% at 2 years, 2–21% at 4–6 years, and 23% at 10 years. This diminishes in older or more medically complex patients, likely due to perceived risks [43]. Laminectomy-only patients face higher reoperation risks than those with fusion, but fusion does not reduce long-term reoperation needs. Revisions yield improvements in pain and function, though less than primary surgeries [44,45,46].

According to Shamji et al., in a review of 11 studies of lumbar spinal surgery for spinal stenosis in elderly patients, wound infection rates were about 2% (range 0–5%) [47].

In our investigation, we did not encounter a recurrence at the same level or lumbar instability thanks to the preservation of spinal anatomy, bone, and paraspinal tissue. In comparing surgical outcomes, our study reports a mean hospital stay of 2.64 days, an average surgical time of 47 min, and a low incidence of complications, including a dural tear rate of 5.85% and a reintervention rate of 0.57%. These results are consistent with those reported in the literature for minimally invasive techniques and are favorable when compared to traditional open laminectomy, which has higher complication rates and longer recovery times. In our study, we observed a dural tear rate of 5.85%, which aligns with the lower end of the reported range for traditional open decompressions and minimally invasive techniques [32,34]. Wound infection was recorded for eight patients and treated conservatively with appropriate antibiotics drugs and advanced medications, without the need for surgery. When comparing surgical outcomes, our approach demonstrated significant improvements in clinical metrics. The mean ODI decreased from 52.2% preoperatively to 24.7% postoperatively, and VAS scores for back and leg pain reduced markedly. These improvements are consistent with or superior to those reported in studies of both open and minimally invasive techniques [23,24].

The step-by-step breakdown provides a clear roadmap, facilitating a comprehensive grasp of the procedure. By recognizing and respecting the quadrilateral boundaries, surgeons can navigate with precision, defining the limits of surgical decompression. Undoubtedly, respect for tissues, ligaments, and bony structures, as advocated in our approach, contributes to maintaining spinal stability and reducing postoperative pain. The emphasis on less aggressive surgical manipulation aligns with the contemporary trend toward minimally invasive techniques, promising improved patient outcomes and satisfaction.

### 6.5. Training of Young Surgeons and Progressive Improvement

During the initial phases of postgraduate training, residents are often exposed to complex cases where hands-on experience is crucial for their development. By focusing on a consistent methodology, the learning curve is steep but manageable, allowing residents to develop a deep understanding of the relevant anatomy and surgical principles without being overwhelmed by variability.

To assess the impact of our approach on resident training, we conducted an informal evaluation of resident performance and satisfaction after their first 10 cases under supervision. Residents reported increased confidence and competence with each case, particularly in the identification of key anatomical landmarks and the precise execution of surgical steps. The gradual transition from observational to hands-on participation in the procedure fostered a sense of ownership over the surgical process, which is critical for skill development.

Residents were asked to rate their satisfaction with the learning experience on a scale of 1 to 10 after completing their first 10 procedures. On average, residents rated their satisfaction as 9.2, citing the clarity and reproducibility of the approach as the most valuable aspects. The technique’s well-defined steps allowed residents to focus on mastering one phase at a time, which contributed to a more gradual yet thorough learning experience. Furthermore, residents noted significant improvements in their technical precision and decision-making, especially regarding the skeletalization of the homolateral lamina and the contralateral decompression step.

The main challenge in postgraduate surgical training is the limited time for hands-on experience due to work hour restrictions, which can hinder skill development. Our three-step approach addresses this by offering a clear, structured framework that can be taught incrementally, allowing residents to gradually take on more responsibility under close supervision. This method promotes teamwork, real-time feedback, and understanding of key anatomical landmarks, ensuring safe, progressive learning despite time constraints, and equipping young surgeons with the skills needed for complex procedures.

## 7. Strength and Limitations

This approach is characterized by its structured and sequential design, which enhances both the clarity and the precision of the surgical procedure. The well-defined sequence of steps not only ensures reproducibility but also allows the surgeon to maintain a clear understanding of each phase of the surgery. This structured framework provides the flexibility to make informed decisions at any point during the operation, thus optimizing patient outcomes. Furthermore, the protocol is grounded in a robust anatomical understanding, which is a fundamental prerequisite for any successful surgical intervention.

The meticulous identification of the anatomical trapezoid and systematic adherence to the three surgical steps can serve as a robust foundation and a valuable exercise, especially for residents or young spinal surgeons, providing a secure and educational framework for addressing lumbar spinal stenosis.

Standardizing and simplifying the procedure into clear, step-by-step instructions allows instructors to guide less experienced surgeons with confidence. This structured approach ensures that more seasoned surgeons can maintain control throughout the operation, minimizing risks to the patient and avoiding unnecessary delays. Each stage of the process is designed to be as safe as possible, even when a trainee is involved.

However, the retrospective design introduces inherent biases, such as selection and information biases, which may affect the generalizability of our findings. Additionally, the variability in follow-up durations (12–48 months) could influence the consistency of long-term outcome assessments. To further validate our findings and minimize these limitations, future research should include prospective, randomized controlled trials to provide more definitive evidence on the efficacy and safety of this approach.

## 8. Conclusions

In the realm of lumbar spinal stenosis, our three-step surgical approach, meticulously designed and rooted in anatomical insights, stands as an invaluable educational tool for junior and senior resident and young spine surgeons. This structured methodology, emphasizing the significance of anatomical landmarks and tissue preservation, caters to the educational needs of young surgeons.

## Figures and Tables

**Table 1:** Demographic data and follow-up.

Demographic Data	N. of Patients	Other Data	
Patients	530	Average Age	67 years (±9.04)
Employed/non-employed	262/268	Male/Female Ratio	262/268
Smokers/Non-smokers	162/368	Mean BMI	26.88 (±4.3)
Arterial Hypertension	273	Mean Follow-up	12–48 months
Previous orthopedic surgery	95		
Fibromyalgia	109		

**Table 2:** Levels and surgical data.

Lumbar Stenosis Treated	N. of Levels	Lumbar Stenosis Treated	N. of Levels	Surgical Data	
Total lumbar patients	530	L5S1	87	Average wound size	4.61 cm (±1.68)
Total lumbar levels		L4L5	360	Mean surgical time	47 min (±19.4)
Single level	325	L3L4	234	Average hospital stay	2.64 days (±0.78)
Double level	157	L2L3	92	Average wound size	4.51 cm (±1.58)
Triple level	39	L2L1	13		
Quadruple level	9	D12D11	1		
		D11D10	1		
		D10D9	1		

**Table 3:** Complications.

Intraoperative Complications	N. of Complications	%	Postoperative Complications	N. of Complications	%
Dural tear	32	6.04	Recurrence	0	0
Atrial fibrillation	1	0.19	Instability	0	0
		0.11	Subcutaneous hematoma	3	0.57
		1.96	Thromboembolism	1	0.19
			Anemia	1	0.19
			Amaurosis left eye	1	0.19
			Wound infection	8	1.52

**Table 4:** Pre-operative and post-operative patient assessment.

Patient Assessment	Pre-Operative	Post-Operative
ODI (Oswestry Disability Index)	52.2% (±18.6)	24.7% (±18.7)
VAS (Visual Analog Scale) Leg Pain	8.01 (±1.4)	3.1 (±2.5)
VAS (Visual Analog Scale) Back Pain	8.01 (±1.37)	2.2 (±2.3)
EQ-5D (EuroQuality of life5 Dimensions)	0.377 (±0.211)	0.684 (±0.233)

**Table 5:** Dimension of bone landmark in the surgical field.

Surgical Triangle Dimension	Characteristics/Borders	Average Length (mm)	Standard Deviation
First side	from the caudal to the cranial point of the base of the spinous process	16.6	2.02
Second Side	from the cranial point of the base of the spinous process to the medial margin of the superior articular process	20.3	1.28
Third Side	from the medial margin of the superior articular process to the medial margin of the inferior articular process	26.5	2.06
Fourth Side	from the caudal point of the base of the spinous process to the medial margin of the inferior articular process	21.5	1.86
Area of drilled bone	Trapezoidal area	437.4 mm^2^	40.4

## Data Availability

The data presented in this study are available on request from the corresponding author.

## References

[1] Sobański D., Staszkiewicz R., Stachura M., Gadzieliński M., Grabarek B.O. (2023). Presentation, Diagnosis, and Management of Lower Back Pain Associated with Spinal Stenosis: A Narrative Review. Med. Sci. Monit..

[2] D’Antonio N.D., Lambrechts M.J., Trenchfield D., Sherman M., Karamian B.A., Fredericks D.J., Boere P., Siegel N., Tran K., Canseco J.A. (2023). Patient-Specific Risk Factors Increase Episode of Care Costs After Lumbar Decompression. Clin. Spine Surg. A Spine Publ..

[3] Katz J.N., Zimmerman Z.E., Mass H., Makhni M.C. (2022). Diagnosis and Management of Lumbar Spinal Stenosis: A Review. JAMA.

[4] Witiw C.D., O’Toole E., Richard W.H. (2022). Cervical, Thoracic, and Lumbar Stenosis. Youmans and Winn Neurological Surgery.

[5] Jang J.N., Song Y., Kim J.W., Kim Y.U. (2023). Comparison of Ligamentum Flavum Thickness between Central and Lateral Lesions in a Patient with Central Lumbar Spinal Canal Stenosis. Medicine.

[6] Sudhir G., Vignesh Jayabalan S., Gadde S., Venkatesh Kumar G., Karthik Kailash K. (2019). Analysis of Factors Influencing Ligamentum Flavum Thickness in Lumbar Spine—A Radiological Study of 1070 Disc Levels in 214 Patients. Clin. Neurol. Neurosurg..

[7] Quattrocchi C.C., Alexandre A.M., Pepa G.M.D., Altavilla R., Zobel B.B. (2011). Modic Changes: Anatomy, Pathophysiology and Clinical Correlation. Acta Neurochir. Suppl..

[8] Aleksić V., Todorović J., Miladinović N., Aleksić N., Bogosavljević V., Đurović M., Kocić S., Aleksić R., Joković M. (2023). Ligamentum Flavum Analysis in Patients with Lumbar Discus Hernia and Lumbar Spinal Stenosis. Sci. Rep..

[9] Porter R.W., Ward D. (1992). Cauda Equina Dysfunction. The Significance of Two-Level Pathology. Spine (Phila Pa 1976).

[10] Sun C., Zhang H., Wang X., Liu X. (2020). Ligamentum Flavum Fibrosis and Hypertrophy: Molecular Pathways, Cellular Mechanisms, and Future Directions. FASEB J..

[11] La Rocca G., Galieri G., Mazzucchi E., Pignotti F., Orlando V., Pappalardo S., Olivi A., Sabatino G. (2024). The Three-Step Approach for Lumbar Disk Herniation with Anatomical Insights Tailored for the Next Generation of Young Spine Surgeons. J. Clin. Med..

[12] Goldberg J.L., Moss N., Virk M.S., Fu K.-M.G., Winn H.R. (2022). Spinal Anatomy. Youmans and Winn Neurological Surgery.

[13] Galieri G., Mazzucchi E., Pignotti F., Rinaldi P., De Santis V., La Rocca G., Sabatino G. (2022). Lumbo-Sacral Pedicular Aplasia Diagnosis and Treatment: A Systematic Literature Review and Case Report. Br. J. Neurosurg..

[14] Yoshiki T., Shuichi M., James D., Disk K., Winn H.R. (2022). Degeneration and Regeneration. Youmans and Winn Neurological Surgery.

[15] Hermansen E., Austevoll I.M., Hellum C., Storheim K., Myklebust T.Å., Aaen J., Banitalebi H., Anvar M., Rekeland F., Brox J.I. (2022). Comparison of 3 Different Minimally Invasive Surgical Techniques for Lumbar Spinal Stenosis: A Randomized Clinical Trial. JAMA Netw. Open.

[16] Overdevest G.M., Jacobs W., Vleggeert-Lankamp C., Thomé C., Gunzburg R., Peul W. (2015). Effectiveness of Posterior Decompression Techniques Compared with Conventional Laminectomy for Lumbar Stenosis. Cochrane Database Syst. Rev..

[17] Rajasekaran S., Thomas A., Kanna R.M., Prasad Shetty A. (2013). Lumbar Spinous Process Splitting Decompression Provides Equivalent Outcomes to Conventional Midline Decompression in Degenerative Lumbar Canal Stenosis: A Prospective, Randomized Controlled Study of 51 Patients. Spine (Phila Pa 1976).

[18] Ruetten S., Komp M., Merk H., Godolias G. (2009). Surgical Treatment for Lumbar Lateral Recess Stenosis with the Full-Endoscopic Interlaminar Approach versus Conventional Microsurgical Technique: A Prospective, Randomized, Controlled Study. J. Neurosurg. Spine.

[19] Costa F., Alves O.L., Anania C.D., Zileli M., Fornari M. (2020). Decompressive Surgery for Lumbar Spinal Stenosis: WFNS Spine Committee Recommendations. World Neurosurg. X.

[20] Morales A., El Chamaa A., Mehta S., Rushton A., Battié M.C. (2024). Depression as a Prognostic Factor for Lumbar Spinal Stenosis Outcomes: A Systematic Review. Eur. Spine J..

[21] Murphy M.E., Gilder H., Maloney P.R., McCutcheon B.A., Rinaldo L., Shepherd D., Kerezoudis P., Ubl D.S., Crowson C.S., Krauss W.E. (2017). Lumbar Decompression in the Elderly: Increased Age as a Risk Factor for Complications and Nonhome Discharge. J. Neurosurg. Spine.

[22] Knutsson B., Michaëlsson K., Sandén B. (2013). Obesity Is Associated with Inferior Results after Surgery for Lumbar Spinal Stenosis: A Study of 2633 Patients from the Swedish Spine Register. Spine (Phila Pa 1976).

[23] Ghobrial J., Gadjradj P., Harhangi B., Dammers R., Vleggeert-Lankamp C. (2022). Outcome of Non-Instrumented Lumbar Spinal Surgery in Obese Patients: A Systematic Review. Br. J. Neurosurg..

[24] Merrill R.K., Zebala L.P., Peters C., Qureshi S.A., McAnany S.J. (2018). Impact of Depression on Patient-Reported Outcome Measures After Lumbar Spine Decompression. Spine (Phila Pa 1976).

[25] Mazzucchi E., La Rocca G., Cusumano D., Bazzu P., Pignotti F., Galieri G., Rinaldi P., De Santis V., Sabatino G. (2023). The Role of Psychopathological Symptoms in Lumbar Stenosis: A Prediction Model of Disability after Lumbar Decompression and Fusion. Front. Psychol..

[26] Canseco J.A., Karamian B.A., Minetos P.D., Paziuk T.M., Gabay A., Reyes A.A., Bechay J., Xiao K.B., Nourie B.O., Kaye I.D. (2022). Risk Factors for 30-Day and 90-Day Readmission After Lumbar Decompression. Spine (Phila Pa 1976).

[27] Ma X.L., Zhao X.W., Ma J.X., Li F., Wang Y., Lu B. (2017). Effectiveness of Surgery versus Conservative Treatment for Lumbar Spinal Stenosis: A System Review and Meta-Analysis of Randomized Controlled Trials. Int. J. Surg..

[28] Wei F.L., Zhou C.P., Liu R., Zhu K.L., Du M.R., Gao H.R., Wu S.D., Sun L.L., Yan X.D., Liu Y. (2021). Management for Lumbar Spinal Stenosis: A Network Meta-Analysis and Systematic Review. Int. J. Surg..

[29] Deyo R.A., Mirza S.K., Martin B.I., Kreuter W., Goodman D.C., Jarvik J.G. (2010). Trends, Major Medical Complications, and Charges Associated with Surgery for Lumbar Spinal Stenosis in Older Adults. JAMA.

[30] Li G., Patil C.G., Lad S.P., Ho C., Tian W., Boakye M. (2008). Effects of Age and Comorbidities on Complication Rates and Adverse Outcomes after Lumbar Laminectomy in Elderly Patients. Spine (Phila Pa 1976).

[31] Sobottke R., Aghayev E., Röder C., Eysel P., Delank S.K., Zweig T. (2012). Predictors of Surgical, General and Follow-up Complications in Lumbar Spinal Stenosis Relative to Patient Age as Emerged from the Spine Tango Registry. Eur. Spine J..

[32] Suzuki A., Nakamura H. (2022). Microendoscopic Lumbar Posterior Decompression Surgery for Lumbar Spinal Stenosis: Literature Review. Medicina.

[33] Zhuang H.X., Guo S.J., Meng H., Lin J.S., Yang Y., Fei Q. (2023). Unilateral Biportal Endoscopic Spine Surgery for Lumbar Spinal Stenosis: A Systematic Review and Meta-Analysis. Eur. Rev. Med. Pharmacol. Sci..

[34] Zhang J., Liu T.F., Shan H., Wan Z.Y., Wang Z., Viswanath O., Paladini A., Varrassi G., Wang H.Q. (2021). Decompression Using Minimally Invasive Surgery for Lumbar Spinal Stenosis Associated with Degenerative Spondylolisthesis: A Review. Pain. Ther..

[35] Fourney D.R., Dettori J.R., Norvell D.C., Dekutoski M.B. (2010). Does Minimal Access Tubular Assisted Spine Surgery Increase or Decrease Complications in Spinal Decompression or Fusion?. Spine (Phila Pa 1976).

[36] Alhaug O.K., Dolatowski F., Austevoll I., Mjønes S., Lønne G. (2023). Incidental Dural Tears Associated with Worse Clinical Outcomes in Patients Operated for Lumbar Spinal Stenosis. Acta Neurochir..

[37] Takahashi Y., Sato T., Hyodo H., Kawamata T., Takahashi E., Miyatake N., Tokunaga M. (2013). Incidental Durotomy during Lumbar Spine Surgery: Risk Factors and Anatomic Locations: Clinical Article. J. Neurosurg. Spine.

[38] Tenhoeve S.A., Karsy M. (2023). Lumbar Epidural Hematoma as a Rare Complication From Minimally Invasive Lumbar Decompression. Cureus.

[39] Soejima Y., Arizono T., Bekki H., Inokuchi A., Izumi T., Imamura R., Hamada T., Nakamura K., Sakai M., Yoshimoto M. (2022). Factors Affecting Postoperative Spinal Epidural Hematoma and the Optimal Order of Vertebral Body Decompression in Multivertebral Microendoscopic Laminectomy. Cureus.

[40] Hohenberger C., Zeman F., Höhne J., Ullrich O.W., Brawanski A., Schebesch K.M. (2020). Symptomatic Postoperative Spinal Epidural Hematoma after Spinal Decompression Surgery: Prevalence, Risk Factors, and Functional Outcome. J. Neurol. Surg. A Cent. Eur. Neurosurg..

[41] Leonardi M.A., Zanetti M., Saupe N., Min K. (2010). Early Postoperative MRI in Detecting Hematoma and Dural Compression after Lumbar Spinal Decompression: Prospective Study of Asymptomatic Patients in Comparison to Patients Requiring Surgical Revision. Eur. Spine J..

[42] Bekki H., Arizono T., Inokuchi A., Imamura R., Hamada T., Oyama R., Hyodo Y., Kinoshita E., Kido M. (2021). Risk Factors for Incidence of Postoperative Spinal Epidural Hematoma Following Multilevel Microendoscopic Laminectomy. Spine Surg. Relat. Res..

[43] Deyo R.A., Martin B.I., Kreuter W., Jarvik J.G., Angier H., Mirza S.K. (2011). Revision Surgery Following Operations for Lumbar Stenosis. J. Bone Jt. Surg. Am..

[44] Radcliff K., Curry P., Hilibrand A., Kepler C., Lurie J., Zhao W., Albert T.J., Weinstein J. (2013). Risk for Adjacent Segment and Same Segment Reoperation after Surgery for Lumbar Stenosis: A Subgroup Analysis of the Spine Patient Outcomes Research Trial (SPORT). Spine (Phila Pa 1976).

[45] Lang Z., Li J.S., Yang F., Yu Y., Khan K., Jenis L.G., Cha T.D., Kang J.D., Li G. (2018). Reoperation of Decompression Alone or Decompression plus Fusion Surgeries for Degenerative Lumbar Diseases: A Systematic Review. Eur. Spine J..

[46] Jung J.M., Chung C.K., Kim C.H., Choi Y., Kim M.J., Yim D., Yang S.H., Lee C.H., Hwang S.H., Kim D.H. (2020). The Long-Term Reoperation Rate Following Surgery for Lumbar Stenosis: A Nationwide Sample Cohort Study With a 10-Year Follow-Up. Spine (Phila Pa 1976).

[47] Shamji M.F., Mroz T., Hsu W., Chutkan N. (2015). Management of Degenerative Lumbar Spinal Stenosis in the Elderly. Neurosurgery.

